# In vivo estimation of target registration errors during augmented reality laparoscopic surgery

**DOI:** 10.1007/s11548-018-1761-3

**Published:** 2018-04-16

**Authors:** Stephen Thompson, Crispin Schneider, Michele Bosi, Kurinchi Gurusamy, Sébastien Ourselin, Brian Davidson, David Hawkes, Matthew J. Clarkson

**Affiliations:** 1 Wellcome/EPSRC Centre for Interventional and Surgical Sciences, University College London, London, UK; 2 Division of Surgery and Interventional Science, University College London, London, UK

**Keywords:** Image-guided surgery, Augmented reality, Liver, Validation, Error measurement, Laparoscope

## Abstract

**Purpose:**

Successful use of augmented reality for laparoscopic surgery requires that the surgeon has a thorough understanding of the likely accuracy of any overlay. Whilst the accuracy of such systems can be estimated in the laboratory, it is difficult to extend such methods to the in vivo clinical setting. Herein we describe a novel method that enables the surgeon to estimate in vivo errors during use. We show that the method enables quantitative evaluation of in vivo data gathered with the SmartLiver image guidance system.

**Methods:**

The SmartLiver system utilises an intuitive display to enable the surgeon to compare the positions of landmarks visible in both a projected model and in the live video stream. From this the surgeon can estimate the system accuracy when using the system to locate subsurface targets not visible in the live video. Visible landmarks may be either point or line features. We test the validity of the algorithm using an anatomically representative liver phantom, applying simulated perturbations to achieve clinically realistic overlay errors. We then apply the algorithm to in vivo data.

**Results:**

The phantom results show that using projected errors of surface features provides a reliable predictor of subsurface target registration error for a representative human liver shape. Applying the algorithm to in vivo data gathered with the SmartLiver image-guided surgery system shows that the system is capable of accuracies around 12 mm; however, achieving this reliably remains a significant challenge.

**Conclusion:**

We present an in vivo quantitative evaluation of the SmartLiver image-guided surgery system, together with a validation of the evaluation algorithm. This is the first quantitative in vivo analysis of an augmented reality system for laparoscopic surgery.

## Introduction

Laparoscopic surgery for liver resection is in general preferable to open surgery, due to the significant reduction in post-operative pain and scarring [7]. Currently only a minority of patients at specialist hospitals undergoes laparoscopic resection. One reason for the low rate of laparoscopic resection is the difficulty surgeons have in identifying key anatomy through a laparoscopic camera and monitor [4]. This can be addressed by introducing external images to the procedure, known as image-guided surgery (IGS). A recent review [5] describes the state of the art of laparoscopic IGS. In most cases Augmented Reality (AR), where a model is overlaid directly on the laparoscopic video, is avoided due to the difficulty in creating a well aligned overlay on a deforming and mobile organ. One approach is to show a solid model derived from pre-operative Computed Tomography (CT) next to the surgical scene. Whilst the model may be orientated to match the surgical scene, it is up to the surgeon to identify the final correspondence between the model and the video. The first reported use of an AR overlay in laparoscopic liver surgery is reported by [10] making the case for the benefits of an AR laparoscopic system. We developed the “SmartLiver” IGS system to show the liver model overlaid on the video feed from a laparoscope. This spares the surgeon some cognitive load; however, it raises questions in terms of perception and interpretation of errors.

**Fig. 1:**
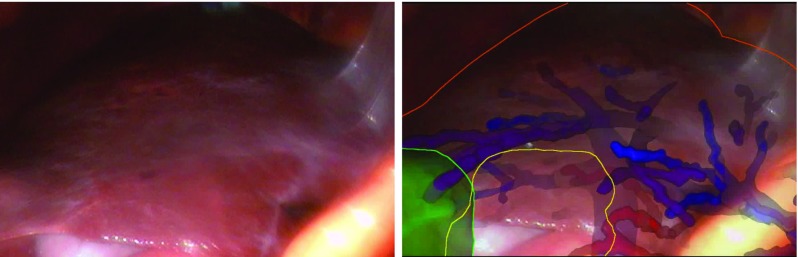
The right liver lobe as seen through the laparoscopic camera, left image. The right image shows the same scene augmented using the SmartLiver system. The outline of the liver, shown as an orange line, can be compared to the visible liver outline. The mismatch gives an estimate of the accuracy of overlay for non-visible vessels, veins (blue and purple) and arteries (red). Also visible is the gall bladder (yellow outline) and a tumour (green)

In any AR system, there will be misalignment between the overlay and what is visible on the screen. Furthermore, it must remain the responsibility of the surgeon to interpret and act upon any apparent error. To enable this, we have implemented advanced visualisation algorithms, to allow the surgeon to rapidly identify AR overlay errors. Figure 1 shows an in vivo overlay using our system. A key feature of the overlay is that we have maintained a projected 2D outline of the liver, which can be compared to the visible anatomy. The outline enables an estimate of the accuracy of any overlaid non-visible anatomy.

## Background

One reason for the slow progress of laparoscopic IGS is a lack of a realistic approach to the measurement and interpretation of alignment errors. In contrast to orthopaedics or neurosurgery, the anatomy of the abdomen is mobile, so IGS using rigid registration may suffer significant localised errors. It is theoretically possible to use deformable registration and motion models [17]; however, this adds complexity, and makes it harder for the surgeon to interpret the system’s performance. Breath hold or gating can also be used to improve the apparent accuracy, at a cost in usability.

Collins et al. [9] investigate the effect of variation in surface reconstruction protocol on rigid and non-rigid surface-based registration. They show that a system using rigid registration can be expected to have registration errors around 10 mm, while deformable registration can get down to approximately 6 mm. These figures are also in agreement with our results.

Kang et al. [13] propose an AR laparoscopic system that avoids some of the problems of soft tissue motion and deformation between scan and surgery by only using intra-operatively acquired ultrasound images. They report errors of approximately 3 mm for their ultrasound only AR system. The primary source of errors in such a system will be tracking and calibration errors, again providing a useful comparison with our system.

Hayashi et al. [12] present a novel registration method for gastric surgery, using subsurface landmarks to progressively improve the registration as and when they become visible during resection. They report accuracies around 13 mm, which is similar to our best achieved accuracy of 12 mm. Interestingly they report that their surgeons believe the system would become useful at accuracies of 10 mm, as the surgeon should be able to mentally compensate for the residual registration errors caused by deformation and motion.

Amir-Khalili et al. [1] propose displaying contours showing uncertainty around the displayed targets. Alternatively, Pratt el al. [15] overlay a wire-frame of the organ surface. In our experience, these approaches are too visually cluttered for liver surgery, hence our proposed use of outline rendering. Communication of alignment errors gets harder when deformable registration is used. Bano et al. [3] show two results relevant to our study in their pre-clinical work on using intra-operative C-arm to inform a non-rigid registration of the liver. Firstly, in their porcine model, deformation due to insufflation is a significant source of registration error (around 8 mm). Furthermore, the error measured at internal vessels is significantly higher (by approximately 6 mm) than the error measured at the liver surface.

## Contributions of this Paper

Our proposed method for in vivo estimation of errors uses the visible misalignment of the liver outline (Fig. 1) to infer the misalignment of non-visible target anatomy. In this paper, we define a measure of visible misalignment, re-projection error (RPE), and test the assumption that RPE is a useful predictor of the misalignment of subsurface targets, or target registration error (TRE). In part this can be estimated using the relations between fiducial localisation error (FLE) and TRE originally put forward by Fitzpatrick and West [11]; however, two factors limit the applicability of their approach. Firstly, the FLE of individual in vivo landmarks are not independent random variables, as they will all be influenced by systematic errors in calibration and tracking of the laparoscope and tissue motion. Independence of FLE is a key assumption of [11] and derived works; therefore, use of these relationships when the assumption is not true can significantly underestimate TRE [19]. Secondly, in our calculation of RPE, errors normal to the camera lens are effectively discarded, because they cannot be estimated from a 2D image. This creates a non-linear transformation from 3D misalignment errors to 2D RPE. Therefore, it is not clear that RPE can be safely used as a proxy for FLE.

In our pre-clinical work, only point landmarks were used for validation [21]; however, during our ongoing in vivo validation we have found it extremely difficult to identify point landmarks on the human liver. In general, the landmarks we have been able to use are concentrated around the high curvature points close to the falciform ligament. In contrast, it is possible to identify line landmarks across the entire visible edge of the liver. To enable validation of the system in vivo, we have therefore developed a novel algorithm to measure RPE using both point and line landmark features.

With this paper, we make three important and novel contributions. We test the validity of using RPE derived from point and landmark features to estimate subsurface TRE, in so doing we enable the translation from pre-clinical to clinical research. Secondly, the algorithm is applied to 9 in vivo cases, to our knowledge this is the first attempt at a quantitative evaluation of a liver AR IGS system on multiple patients. Lastly we describe the ongoing development of the SmartLiver system, including the use of a novel rendering engine to enable in vivo visualisation of misalignment errors and an improved user interface.

## Materials and methods

### SmartLiver surgery workflow using surface-based registration

The SmartLiver system hardware consists of a workstation PC and a Polaris Spectra^1^ optical tracking system, mounted on a custom built trolley with an un-interruptible power supply. The PC runs custom software based on the NifTK software platform [8]. The PC includes an NVIDIA SDI capture card and an NVIDIA K6000 GPU. In theatre, the system stands next to the laparoscopic stack, allowing the surgeon to see an augmented reality overlay near their existing line of sight.

**Fig. 2:**
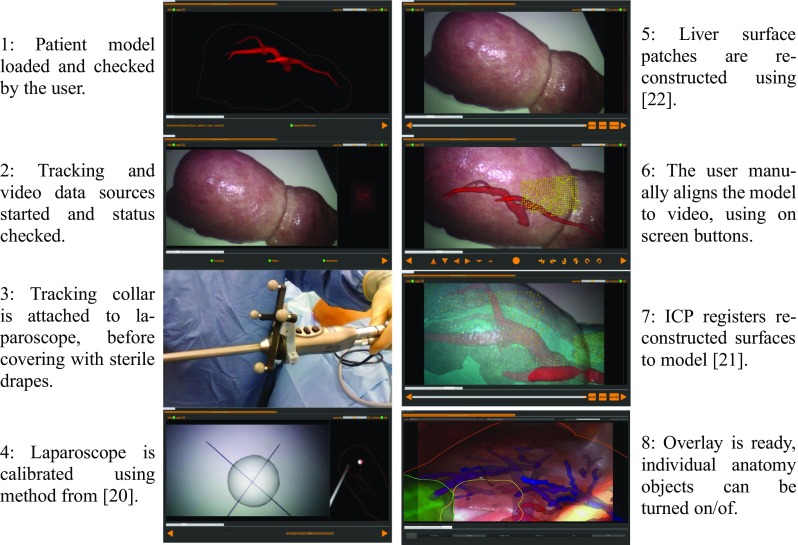
Flow diagram of the SmartLiver IGS software. The user runs through 7 tabbed screens, moving from system initialisation to registration and overlay. To provide the clearest possible images, we have used a mixture of images from clinical use (panels 3, 4, and 8) and phantom testing (panels 1, 2, 5, 6, 7)

Figure 2 shows the software flowchart and user interface from start up to augmented reality overlay. Up until the patient being ready for surgery, set-up time does not impact on total theatre time. Once the patient is anaesthetised and ready for surgery, time is critical, hence the need for a well-defined work flow and simple user interface. The in vivo data reported in this paper was gathered using earlier versions of the user interface. Because the user interface was often difficult to use the quality of any registrations performed in theatre is highly variable, as will be seen in the results.

Steps 6 and 7 in Fig. 2 define the transform from model space to world space, henceforth denoted $T_{M2W}$. Once $T_{M2W}$ is estimated the surgeon can refer to the augmented reality display, to localise subsurface anatomy. Steps 6 and/or 7 can be repeated to give a new estimate of $T_{M2W}$ if the liver moves significantly. The visualisation (Fig. 1) shows visible anatomy as a 2D outline and internal anatomy as a depth fogged surface model. Visualisation is implemented using the Visualisation Library.^2^ The surgeon can use the mismatch between the visible and projected outlines to make a rapid assessment of the system accuracy. Analysis of registration accuracy was performed after surgery, using data saved during surgery. These data consist of video and tracking data recorded throughout the procedure, calibration data for the laparoscope, and any estimates of $T_{M2W}$ from in-theatre registrations.

### Estimation of re-projection error

Errors in augmented reality can be estimated in some applications where features are visible in both the video and in the projected model. This approach was described in our previous publication [21] on pre-clinical and phantom data and is extended here.

Landmark points on the CT derived model and on the video data were manually identified by a surgeon who had been trained in the use of our software. Point and line picking on the model was done using NifTK [8], utilising MITK’s [14] point set interaction plugin. We wrote a custom point and line picking application for the video data, which now forms part of the NifTK software suite. The software scans through a recorded video file stopping every *n* frames where *n* is set by the user, typically between 25 and 100 frames, depending on the length of the recorded video. The software finds the nearest (in time) tracking data to the video frame and checks the timing difference. If the tracking data are from within 20 ms of the video frame the user is shown a pair of still images from the left and right channels. If the timing difference in greater than 20 ms the frame is skipped.

When presented with the two still images the user is able to click on either of them to define visible landmarks. The user can toggle between point and line selection mode. The landmarks correspond to those selected on the patient model. Landmarks not visible in a given frame are simply excluded.

We have written another application to determine RPE using the landmark points, the camera calibration, the camera tracking data, and $T_{M2W}$. For each frame of video where landmark points have been picked, the error in pixels between the picked landmark and its projected location on the model is calculated. Landmarks that do not project onto the screens visible area are excluded from the analysis.

Representing errors in pixels is problematic for two reasons. Firstly, it has no physical meaning, the surgeon is interested in how the system errors compare with anatomy, for example the smallest vessel size that can be safely cut through and cauterised (approx 3 mm). Secondly, it makes no account of the distance of the object from the camera. If the geometric error (in mm) remains the same, the pixel error will increase as the camera gets closer to the object. To counter this problem, we “re-project” the on-screen errors onto a plane parallel to the camera frame at the distance of the corresponding model feature. The distance between the two points on this plane can be measured in millimetres. Because the on-screen point is back projected onto a plane passing through the corresponding model point, there is no error in the direction normal to the camera plane (the z direction).

**Fig. 3:**
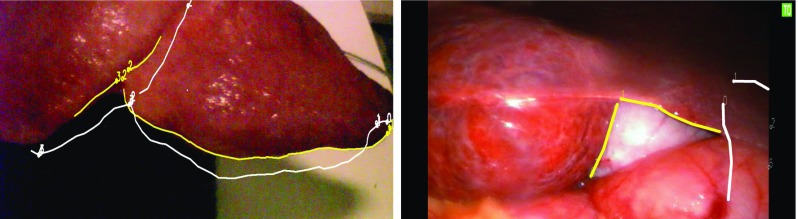
Example projection using the surface features on the phantom (left) and on in vivo data. The on-screen features (shown in yellow) are defined on the recorded video images. The projected features (in white) are projected from the model using the estimated $T_{M2W}$

The above approach was used on phantom and pre-clinical data using landmark points [21]. However, we found it was difficult to identify corresponding landmark points for in vivo data. Specifically, it was very difficult to find point features away from the centre of the liver (near the falciform ligament). In contrast, line features, such as the liver edges, can be identified across the entire liver and used by the surgeon to assess accuracy. Therefore, the methodology was extended to allow the use of line features on the liver surface. The user defines lines as a set of discrete vertices on both the model and the video. When calculating errors, the lines on the video images are treated as a set of discrete vertices, whilst linear interpolation between vertices is used on the model. Figure 3 shows examples of line and point features identified on phantom and in vivo data. The question of how to measure RPE using lines is more ambiguous than for points. We use the following algorithm:

1. Define uniquely identifiable points and lines (points connected by straight segments) on the CT derived liver surface model.
2. For a given video frame, mark any visible points and lines. Partial lines may be used, i.e. there is no requirement that the whole line is visible on the video frame.
3. Each line vertex on the image is re-projected along a ray through the camera’s origin.
4. Transform model features to the camera lens’ coordinate system using $T_{M2W}$ and the world to camera transform.
5. For each ray, find the closest point (*x*) on the corresponding model line.
6. Define a plane (*p*) parallel to the camera image plane passing though (*x*).
7. Compute the distance between point *x* and the intersection of the ray with plane *p*.
8. The mean distance for all vertices of the re-projected line is the RPE for that feature.

### Experiment 1: correlation of TRE and RPE on a liver phantom

**Fig. 4:**
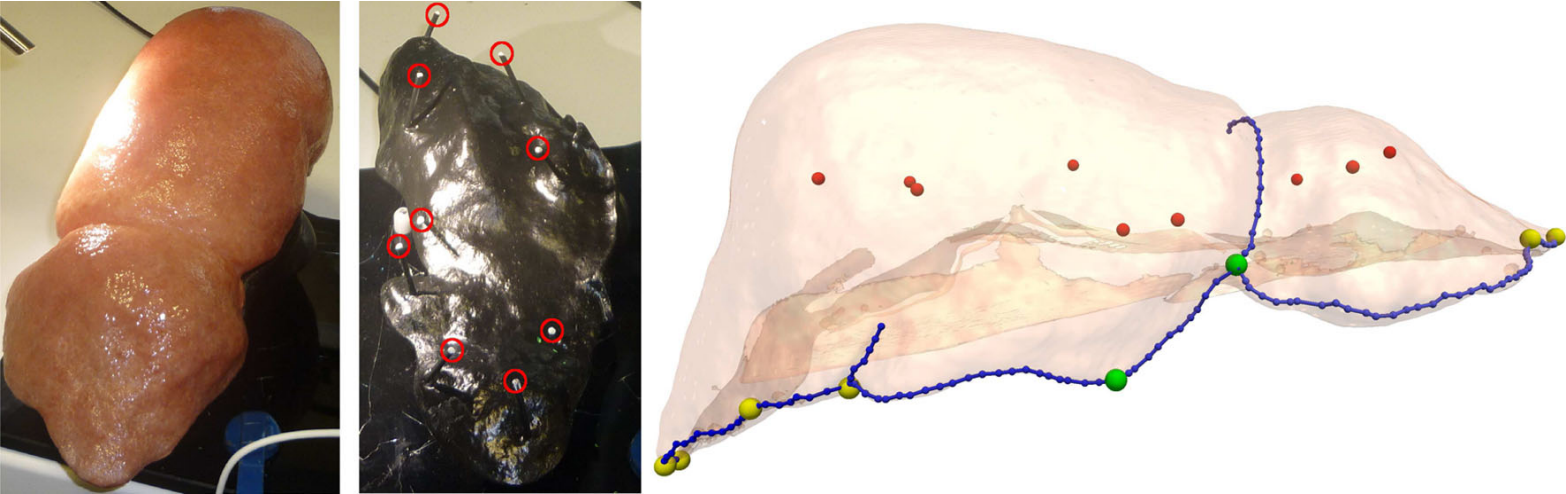
The silicone liver phantom used for validation. The exterior (left) is representative in appearance and geometry of an adult male liver. The internal pins (centre, highlighted in red) secure the liver phantom and act as subsurface landmarks for the measurement of TRE. The right-hand image shows the relative positions of the subsurface targets and surface landmarks. The 9 targets are shown in red, the 6 peripheral surface point landmarks are shown in yellow, the 2 central landmarks in green and the 9 line features in blue

The assumption that RPE can be used to estimate TRE is fundamental to the utility of our proposed IGS system. We test this assumption here. To estimate the system’s accuracy in localising subsurface landmarks a custom made silicone phantom was utilised,^3^ see Fig. 4.

The shape of the phantom was taken from a CT scan of an adult male liver. The external appearance was designed to be representative of a healthy adult liver to enable testing of computer vision algorithms on the phantom [21]. The outer part of the liver phantom is made from flexible silicone and can be repeatably mounted on a set of 9 rigid pins inserted into a moulded epoxy base, see Fig. 4. This configuration enables future work on deformable registration, by utilising bases with different pin geometry.

For this paper, we treat the 9 positioning pins as subsurface targets, so the accuracy of a given estimate of $T_{M2W}$ can be assessed by removing the flexible liver phantom and measuring the pin head locations. This method depends on the repeatability of the positioning of the flexible liver phantom on the base, which was checked by taking 2 CT scans, with the liver phantom removed and replaced between each scan. The CT scans were then aligned using the pin head positions and the alignment of the liver phantom surfaces compared visually. No significant misalignment was observed.

The model to world transform, $T_{M2W}$, could be found by using a separate tracked pointer to locate the pin heads in world space; however, we do not use this method as it gives an inaccurate measure of the overlay errors observed in the SmartLiver system. Use of a separate pointer results in errors in the hand-eye and left to right lens calibration of the stereo laparoscope showing up as a linear offset. The SmartLiver system avoids the need for a highly accurate hand-eye calibration by performing all localisation and overlay in the coordinate system of the laparoscope lens. The liver model is located relative to the laparoscope lens position at some time zero. The model is placed in world coordinates using the hand-eye transform and tracking data. The model is subsequently projected on to the screen using the same hand-eye transform. Provided the laparoscope motion is limited between time zero and the time of AR projection the inaccuracies in the hand-eye calibration largely cancel out. As a clinical laparoscope is constrained by the trocar, we have found this to be the case during pre-clinical and clinical evaluation of the system.

To get a more relevant error measure $T_{M2W}$ is found using stereo triangulation as follows. The pin head positions are manually defined in multiple stereo image pairs taken from a video sequence of the uncovered pin heads. The 3D position of the pin relative to the left camera lens is triangulated using the pixel location in each stereo pair, the two cameras’ intrinsic matrices and right to left lens transform. The triangulated points are placed in world coordinates using the hand-eye and tracking transforms. The result is a point cloud in world space for each pin head. The pin heads defined in the model are registered to the centroids of these point clouds by minimising fiducial registration error (FRE) a per Arun et al. [2]. RPE for this ideal model to world transform (denoted $T_{M2W(i)}$) will not be zero, as errors due to tracking, calibration, and point picking will still be present; however, the RPE will be approximately minimised, giving the surgeon the best possible estimate of the position of the subsurface targets. Therefore, $T_{M2W(i)}$ is assigned a zero TRE. Any other model to world transform for the phantom data set can be described in terms of its TRE relative to $T_{M2W(i)}$.

The experiment we performed consisted of:

1. Identify landmark points and lines in the CT model of the liver phantom.
2. Record a tracked video sequence of the surface of the liver phantom.
3. Remove the silicone phantom, record a tracked video sequence of the subsurface pins.
4. Identify landmark points in both videos, plus lines in the surface videos.
5. Measure the RPE of landmarks (pin heads and surface points and lines) using $T_{M2W(i)}$.

The RPE thus found will be substantially lower than that observed for in vivo data due to the absence of numerous error sources encountered in vivo, most significantly errors due to liver motion and deformation, but also the difficulty in achieving the optimum rigid body registration. Though the sources of error are varied, we make the assumption that their combined effect can be modelled using perturbations of $T_{M2W(i)}$. To create sufficient data to test for correlation between RPE and TRE, we generated 20,000 random perturbations of $T_{M2W(i)}$, and measured the root mean square (RMS) values of RPE and TRE at each.

Random perturbations were defined by 6 independent random variables, 3 translations and 3 rotations. All rotations were about the centroid of the liver phantom. Translations were randomly sampled from a zero mean normal distribution of standard deviation 1.0 mm. Rotations were randomly sampled from a zero mean normal distribution of standard deviation 1.2$^\circ$. The scaling (1.2$^\circ$ per mm) was set so that a translation or rotation of 1 standard deviation results in the same mean absolute displacement across the liver phantom. Rotations and translations were then scaled (using the same scalar for all six vectors) to give a defined normalised Euclidean distance from $T_{M2W(i)}$. Sampling in this way generates perturbations uniformly distributed along each of the 6 degrees of freedom. 1000 random perturbations were generated at each integer value of normalised Euclidean distances from 1 to 20 in. The range of normalised Euclidean distances was set to provide a usable distribution of results at clinically representative RPE.

At each perturbed transform (denoted $T_{M2W(p)}$), TRE and RPE were calculated for each available landmark. RMS values for each measure over multiple landmarks were then calculated and reported. RMS TRE was calculated using Eq. 1, where $X_i$ is the position vector for each of the nine targets (pin heads) in world coordinates.

1 $$\begin{aligned} \mathrm{TRE}_\mathrm{RMS} = \sqrt{\frac{1}{9} \sum ^9_{i=1} (X_i - T_{M2W(p)} X_i)^2} \end{aligned}$$

Three measures of RMS RPE were calculated using different subsets of the surface features shown in Fig. 4. The first uses all 8 available surface point landmarks, the second only uses the 2 point landmarks near the falciform ligament to represent the sort of point features that can be located in vivo. The last measure of RMS RPE uses these 2 point landmarks together with 9 line features, predominantly along the front edge of the liver phantom, representative of the line features that can be located in vivo.

### Experiment 2: evaluation of in vivo data

For in vivo data, there exists no ideal transform as the position of subsurface landmarks remains unknown. However, we were able to collect substantial amounts of in vivo clinical data, following as closely as possible the protocol described in section “SmartLiver surgery workflow using surface-based registration”. To date we have evaluated the accuracy on nine clinical procedures. In each case landmark points were identified in the CT-derived liver model and in several hundred frames of video per patient.

Where available, any model to world transforms, $T_{M2W}$, determined by manual alignment in theatre were used to measure RMS RPE on surface landmarks. Where sufficient point landmarks were available it was also possible to estimate $T_{M2W}$ based on triangulation and registration of surface landmark points. Such landmark-based registration is used by similar liver IGS systems [6, 12], so it makes a useful comparison with our system.

In most cases, we also recorded ex vivo laparoscope calibration data, either of a cross-hair [20] or in earlier cases of a chessboard calibration grid [23]. These calibration data were used to assess the accuracy of the system in theatre in the absence of tissue motion. Chessboard corners or cross-hair centres were manually identified in the video data for tens of frames per data set. These feature were triangulated to world coordinates, and these used to measure re-projection error using the method described in section “Estimation of re-projection error”. Using this method will include errors in picking the points in the video frames, allowing a more direct comparison with the in vivo accuracy, in contrast to reporting the calibration residual errors.

## Results

### Experiment 1: correlation of TRE and RPE on a liver phantom

Video of the liver phantom surface was recorded, imaging the surface point and line landmarks. A total of 2296 stereo images were recorded ($2\times 540\times 1920$ pixels). The laparoscope was moved steadily by hand to try and image each landmark, at an average speed (measured at the lens) of approximately 30 mm/s. A total of 68 images were manually annotated with the positions of point and line landmarks by an experienced research scientist, to give a total of 76 point landmarks and 104 line landmarks. The flexible silicone liver phantom was then removed from its base. A total of 2460 stereo pairs of the securing pin heads were recorded, with the laparoscope again moved steadily around the phantom at a speed of approximately 35 mm/s. A total of 44 frames were manually annotated, giving 87 samples of the pin head positions.

The pin heads picked in the CT model and the pin heads triangulated from the video form two sets of ordered fiducial points, allowing $T_{M2W(i)}$ to be found by minimising FRE as per [2]. The residual FRE was 2.55 mm, suggesting an error in localising each pin head of around 2.89 mm using equation 10 from [11]. The RMS RPE at $T_{M2W(i)}$ was 2.15 mm

**Fig. 5:**
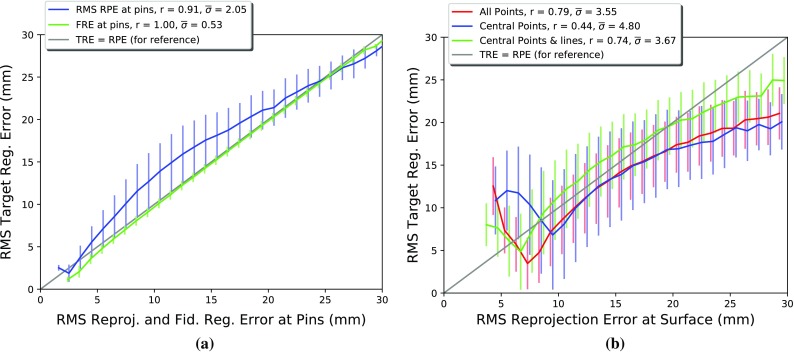
RMS RPE measured on visible features versus RMS TRE measured at the pin heads for the phantom. **a** shows the RMS RPE measured using 9 subsurface pin heads (i.e. with the silicone liver phantom removed from its base.) **b** shows the RMS RPE measured using 3 subsets of features on the on the liver phantom’s surface

Figure 5 plots the distribution of RMS TRE versus RMS RPE and FRE. Each of the 20,000 registrations were binned according to their RMS RPE in 1 mm bins centred around integer values from 1 to 30mm, for each bin the mean and standard deviation of RMS TRE is plotted. Correlation between RMS TRE and RMS RPE or FRE was measured using Pearson’s correlation coefficient (*r*), and the mean standard deviation ($\overline{\sigma }$) over all bins. Figure 5a plots RMS TRE versus RMS RPE and FRE when evaluated on the pin heads themselves. Both RMS RPE and FRE correlate very well with RMS TRE, an unsurprising result given that the measurements are all made on the same features, but confirmation that RPE can be used as a proxy for TRE in the ideal case.

Figure 5b plots RMS TRE versus RMS RPE when RPE is calculated using surface landmark features only, whilst the RMS TRE is measured at the subsurface pin heads. The first (red) line shows the result for when all (8) surface point landmarks are used for measuring RPE, similarly to our pre-clinical results ([21]). In this case, RMS RPE provides a good predictor of RMS TRE with a Pearson’s correlation coefficient of 0.79. This suggests that in cases where surface point landmarks are available over a significant area they provide a useful indicator of subsurface accuracy. The second (blue) line shows a more clinically realistic situation in which only those point landmarks near the falciform ligament are used to calculate RPE. In this case, the correlation coefficient is significantly reduced (to 0.44), and what correlation there is predominantly occurs above RMS RPE of 15 mm, making this measurement of questionable clinical use. The third (green) line in Fig. 5b shows how correlation can be improved by incorporating surface line features, which can be identified in vivo.

### Experiment 2: evaluation of in vivo data

Data from nine patients have been analysed. Acquisitions were made during surgery with the laparoscope being slowly moved by hand. Acquisition time and speed varied, but typically consisted of 1–3 minutes of video with the laparoscope lens moving at around 10–20 mm/s. The number of point and line features used varied between patients. The minimum number of points was 3 and the maximum was 7. The minimum number of lines was 5 and the maximum was 9. An average 469 frames of video data were manually annotated per patient, with a minimum of 80 and a maximum of 1909^4^ frames. Annotation of the video and CT was done by an experienced laparoscopic surgeon. In all cases RPE was measured on a static calibration pattern, on a set of triangulated in vivo point landmarks, and a set of in vivo lines and points registered using point-based registration. The resulting RMS RPE is recorded in the first three numerical columns of Table 1.

The last three numerical columns in Table 1 show the results of registrations performed using the SmartLiver system’s user interface. In four cases, registration was performed during surgery, (Manual Live Alignment), in three different cases manual alignment was performed after surgery on recorded data (Manual Retro. Alignment). Registration using the surface-based Iterative Closest Point (ICP) algorithm was performed once, using surface patches grabbed during surgery. Due to the small sample sizes, we have not performed any statistical comparison of the different registration methods.

As in our pre-clinical work [21], it is useful to analyse the in vivo results in terms of what error sources contribute to the overall error. The bottom 10 rows of Table 1 show which error sources contribute to each result.

**Table 1:** Average RMS RPE errors measured for the human clinical data, classified by what error sources contribute to each error measurement

	Registration method					
	Calib. Error	Triang. Points	Point-Based Reg.	Manual Live Align.	Manual Retro. Align.	ICP Retro. Align.
Average RMS RPE	7.5	10.2	16.3	25.0	19.4	12.3
Standard deviation (Samples)	5.2 (9)	3.3 (9)	3.9 (9)	8.8 (4)	7.4 (3)	− (1)
Contributing errors						
Laparoscope tracking	$\checkmark$	$\checkmark$	$\checkmark$	$\checkmark$	$\checkmark$	$\checkmark$
Laparoscope calibration	$\checkmark$	$\checkmark$	$\checkmark$	$\checkmark$	$\checkmark$	$\checkmark$
Picking points in video	$\checkmark$	$\checkmark$	$\checkmark$	$\checkmark$	$\checkmark$	$\checkmark$
Picking points in CT	–	–	$\checkmark$	$\checkmark$	$\checkmark$	$\checkmark$
Ordered point-based registration	–	–	$\checkmark$	–	–	$\checkmark$
Manual registration	–	–	–	$\checkmark$	$\checkmark$	–
Operating room conditions	–	–	–	$\checkmark$	–	–
ICP surface-based registration	–	–	–	–	–	$\checkmark$
Static deformation (insuflation)	–	–	$\checkmark$	$\checkmark$	$\checkmark$	$\checkmark$
Dynamic deformation (breathing)	–	$\checkmark$	$\checkmark$	$\checkmark$	$\checkmark$	$\checkmark$

Cells containing ticks indicate that a given error source (rows) contributes to total error for a given registration method (columns)

## Discussion

Several tentative conclusions can be drawn from Table 1. The combination of dynamic and static deformation and laparoscope tracking and calibration errors is at least 10 mm. This is the best case accuracy for a laparoscopic IGS utilising optical tracking and a rigid model. There is a slight improvement in RMS RPE for retrospective manual alignment versus in theatre manual alignment, probably due the time pressure and ergonomic compromise present during surgery. The best RMS RPE was found using the surface-based ICP; however, there remain significant challenges to make this process robust.

In vivo results indicate that it is possible to achieve apparent accuracies (RPE) of around 12 mm, which correspond to mean subsurface accuracies around 15 mm (green line in Fig. 5b) with a rigid registration system. Whether such accuracy is clinically useful is currently unknown. The SmartLiver IGS system is at present the only laparoscopic liver surgery system where an augmented reality overlay is attempted routinely. Clinical evaluation is ongoing to try and link accuracy achieved to clinical outcome. Clinical evaluation will also enable an analysis of the most useful way to report errors, i.e. here we report RMS errors, whereas it may be more relevant to focus on the extreme values. Anecdotally, surgeons were generally impressed with the overlays achieved, giving encouragement that the system may be useful at its current accuracy level.

Our long-term aim is to develop a clinical guidance system which can reliably achieve accuracies better than 5 mm, in order to allow the surgeon to navigate around vessels of that size. However, this target was set in the absence of an agreed method to measure accuracy, so is somewhat arbitrary. Nonetheless, the results presented here indicate that accuracies better than 10 mm can only be achieved by deformable registration. Deformable registration and breathing motion compensation [16] of the liver has been shown to be technically possible by several groups [9, 17]. This raises the question of how the surgeon interprets alignment errors when the model has been computationally deformed. Further work could compare TRE and RPE over a wider range of liver shapes and incorporating deformable registration. Our proposed approach of using the 2D projected organ outline should continue to allow a rapid in vivo assessment of error.

Our phantom results, Fig. 5, indicate that the addition of line landmark features results in a smaller RPE for the same TRE. This is likely due to the greater degree of freedom in matching two lines. In this instance, this has helped bring the RPE values closer to TRE; however, this result may be specific to the geometry tested. Further work is required to determine whether this is true in a more general case.

Based on the phantom results, positive correlation between RPE measured at the surface and TRE at subsurface landmarks breaks down below RMS RPE of around 6 mm when using points and lines and 10 mm when using central points only. The main cause of this is likely to be the geometric relationship between the position of surface landmarks and the subsurface targets. In theory, the same rules that govern the design of fiducial markers and tracked instruments [11, 19, 24] can inform the ideal choice of in vivo surface landmarks to use for error estimation. We have begun work [18] looking at the what surface features provide the best registration, which could be extended so that the overlay only shows portions of the liver edge to maximise correlation between apparent RPE and TRE.

## Conclusion

We have described some aspects of the in vivo clinical use of the SmartLiver AR IGS system. We have highlighted some of the many challenges involved in the transition from pre-clinical to clinical research in IGS. Not least of these is the need for a clear and well-validated method to determine the in vivo accuracy. The algorithm we have presented, tested, and used should enable the evaluation of the IGS system on a larger patient cohort, potentially showing a correlation between overlay accuracy and clinical outcomes.

## Footnotes

1 Northern Digital Inc. www.ndigital.com.

2 www.visualizationlibrary.org.

3 www.healthcuts.co.uk.

4 Excluding two outliers the minimum number of frames used is 210, and the maximum 462.
